# In Situ Scanning Electron Microscopy Observation of Crack Initiation and Propagation in Hydroxide Films Formed by Steam Coating on Aluminum-Alloy Sheets

**DOI:** 10.3390/ma13051238

**Published:** 2020-03-09

**Authors:** Hongmei Li, Naoki Takata, Makoto Kobashi, Ai Serizawa

**Affiliations:** 1 Department of Materials Process Engineering, Nagoya University, Furo-cho, Chikusa-ku, Nagoya 464-8603, Japan; takata.naoki@material.nagoya-u.ac.jp (N.T.); kobashi.makoto@material.nagoya-u.ac.jp (M.K.); 2 Department of Materials Science and Engineering, Shibaura Institute of Technology, Toyosu, Koto-ku, Tokyo 135-8548, Japan; serizawa@shibaura-it.ac.jp

**Keywords:** steam coating, aluminum alloy, hydroxide film, adhesion, microstructure, in situ observation, bending test

## Abstract

Hydroxide film was formed on 6061 Al-alloy (Al-1.00Mg-0.62Si(wt.%)) sheets by steam coating with the temperature of 220 °C for 24 h. During bending test of the coated specimens, the crack initiation and propagation processes in the hydroxide film were investigated using in situ SEM observations. The hydroxide film formed exhibited a dual-layer structure composed of an inner amorphous layer and an outer polycrystalline γ-AlO(OH)-phase layer. On the compressively strained surface, lateral cracks are preferentially initiated inside the inner amorphous layer, and propagate either inside this layer or on its interface with the outer γ-AlO(OH) layer. Digital image correlation analyses of the in situ observed SEM images suggested that the concentrated tensile strain along the surface normal localized at some parts of the amorphous layer could contribute to the crack initiation. On the tensile-strained surface, a number of cracks were initiated inside the inner amorphous layer along the surface normal and propagate into the outer γ-AlO(OH) layer. No cracks were found along the interface of the amorphous layer with the Al-alloy substrate. As a result, the anticorrosion hydroxide film adhered on the Al sheet after bending deformation. Such strong adhesion contributes to the excellent corrosion resistance of the Al-alloy parts provided by the steam coating.

## 1. Introduction

Aluminum (Al) alloys are extensively used in the automotive industry and engineering applications [1] owing to their high strength-to-weight ratio, good formability, and corrosion resistance. The 6061 alloy (Al–Mg–Si ternary alloy) is very desirable in automotive, construction, and aerospace industries because of its superior mechanical properties provided by the fine precipitates created through heat-treatment processes [2,3]. The T6 heat treatment involving solution heat treatment and subsequent aging is commonly used for improving the strength of alloys. Due to the formation of a stable Al_2_O_3_ barrier, the T6-treated 6061 alloy is protected under atmospheric environments. However, localized and galvanic corrosions can still take place in it under corrosion environments [4]. Although various surface treatment techniques, such as anodic oxidation and chemical conversion, could be used for improving the corrosion resistance of Al alloys, the heavy metal ions and liquid wastes generated in these processes may cause pollution [5,6]. Thus, it is essential to develop an environmentally friendly surface treatment to form anticorrosion films on the surface of alloys.

A chemical-free “steam coating” process has been developed to prepare an anticorrosion film on the surface of Mg alloys [7]. The steam coating creates an anticorrosion film mainly composed of Mg(OH)_2_ and Mg–Al layered double hydroxide, such a film can provide excellent corrosion resistance [7,8,9]. In addition, this process has also been applied to improve the corrosive resistant Al alloys [10], because of the formation of a continuous Zn-rich amorphous layer and Zn–Al layered double hydroxide (LDH) with a dual-layer structure (a continuous amorphous layer on inside and a polycrystalline hydroxide layer on the outside) [11]. In the case of deformable Al-alloy sheets for automotive parts (e.g., vehicle structure, panels, and wheels), the hydroxide film must adhere well on the substrate in the plastic-forming process. We have previously examined the cracking behavior inside the hydroxide film provided by steam coating on Al-alloy sheets under tension and compression by using a conventional bending test [12]. The results indicated that peeling of the hydroxide film did not occur on the highly strained surface of the Al substrate. Hence, the anticorrosion hydroxide film for suppressing corrosion in steam-coated Al-alloy sheets is robust during the plastic-forming process. Such superior adhesive properties are likely due to the dual-layer structure of the hydroxide film. Nevertheless, the detailed film fracture mechanism remains unclear, especially the initiation site and propagation pathway of cracks inside the hydroxide film and their dependency on the strain state (compressive or tensile).

To better understand the reason behind the superior adhesion properties, in this study the crack initiation and propagation were examined by in situ SEM observations of the steam-coated specimens during bending tests. Based on the results, we discuss the microstructural parameters of the hydroxide film that contribute to the high cohesive strength.

## 2. Experimental Procedure

Commercial material Al 6061 T6 alloy in the form of 1.5 mm-thick sheets [13] were used in this present study. The chemical composition of the alloy (Al-1.00Mg-0.62Si-0.28Cu-0.40Fe-0.02Zn (wt.%)) was analyzed by inductively coupled plasma atomic emission spectroscopy (ICP-AES, Hitachi, Fukuoka, Japan). For the bending test, the specimens were prepared from the provided Al-alloy sheets with a dimension of 30 × 5 × 1.5 mm^3^, subsequently processed by steam coating at 220 °C for 24 h in a saturated steam atmosphere using a horizontal autoclave made of stainless steel with a volume of 100 mL. After that, specimens were ultrasonically cleaned in absolute ethanol for 10 min and dried with Ar gas with a purity of 99.998% [10].

For microstructural observation, cross sections of the Al-alloy samples were ion-polished using an argon-ion beam cross-section polisher (CP) at 6 V over for 7 h. The hydroxide films formed by steam coating on the prepared specimens were observed from transverse and normal directions using a JEOL scanning electron microscopy (SEM) and field-emission type SEM (FE–SEM) operated at 20 kV. The orientation distribution of the α-Al matrix was analyzed by an electron backscattered diffraction (EBSD) operating at a step size of 0.5 μm. For transmission electron microscopy (TEM) observation, thin cross-section samples were prepared using a focused ion beam (FIB) combined with a pickup technique [14]. TEM/STEM (scanning transmission electron microscopy) characterizations of the prepared thin cross-section samples were performed with a TEM instrument (JEOL JEM-2100plus, JEOL Ltd, Tokyo, Japan) operated at 200 kV. The elemental distribution maps were analyzed by TEM–energy dispersive X-ray spectroscopy (EDS) at 200 kV.

**Figure 1:**
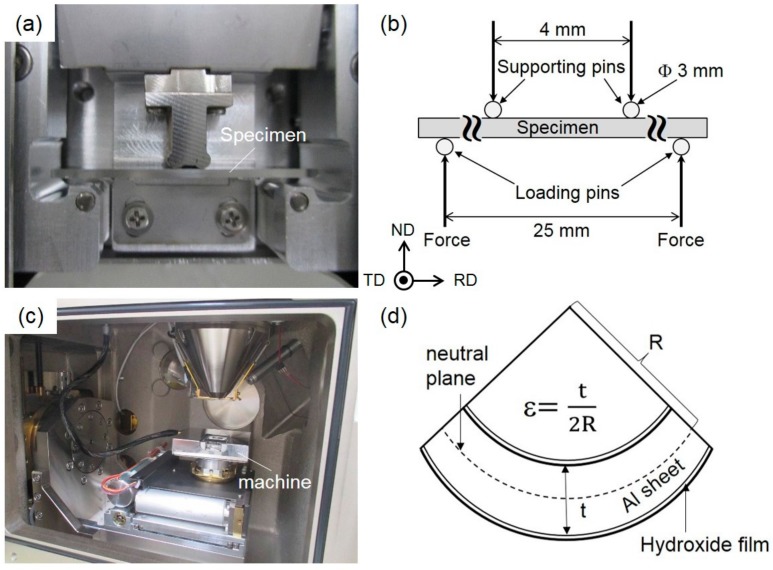
(**a**) Photograph of the specimen set in the miniature-sized bending test machine; (**b**) Schematic geometry used for the 4-points bending test; (**c**) The miniature-sized bending test machine placed in the SEM vacuum chamber for in situ observation; (**d**) Schematic of the bent specimen’s geometry. TD: transverse direction, RD: rolling direction, ND: normal direction, strain: ε, t: specimen thickness, R: radius of curvature.

Load-displacement curves of bent specimens were obtained by 4-points bending tests using a newly designed miniature-sized bending test machine (Figure 1a) at room temperature and a constant displacement rate of 5.8 μm/s. 

The geometry in the 4-points bending test is schematically presented in Figure 1b. The bending test machine loaded with a used specimen was placed inside the vacuum chamber of the SEM instrument (JEOL JSM-6610) for in situ observation of the ion-polished surfaces, as presented in Figure 1c. The strain (*ε*) applied to the surface of the bent specimen was calculated by the following equation:

(1) $$\varepsilon \;=\;\frac{t}{2R}$$

where *t* is the specimen thickness and *R* is the radius of curvature, as schematically shown in Figure 1d. *R* was determined from the low-magnification in situ SEM image during the bending test in the SEM chamber. The local strain distribution around the hydroxide/Al interface in the bent specimens was quantified by digital image correlation (DIC) analysis [15,16] using the in situ observed SEM images at different states of deformation. In the present study, DIC analyses were performed using high-magnification SEM images at a subset size of approximately 81 × 81 pixels.

## 3. Results

### 3.1. Microstructure Observation of Al Substrate and Hydroxide Film

**Figure 2:**
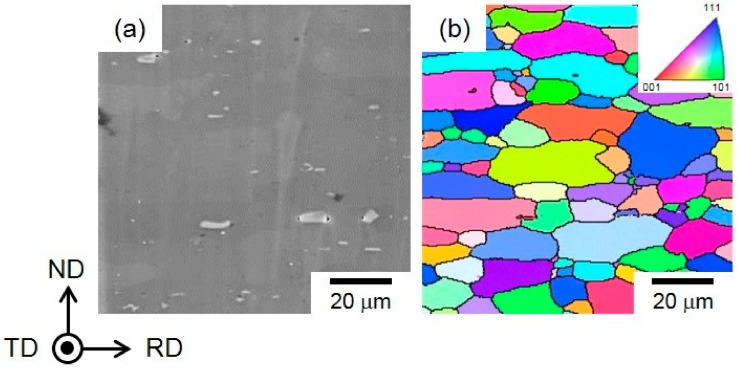
(**a**) Secondary electron image (SEI) and (**b**) corresponding orientation color maps (obtained by electron backscattered diffraction (EBSD) analysis) showing the microstructure of the polished cross section in steam-coated 6061 alloy.

Figure 2 presents secondary electron image (SEI) of the α-Al (face-centered cubic (fcc)) matrix in the steam-coated specimen and corresponding orientation color map (measured by EBSD analyses). In the map, the color code key for the transverse direction (TD) orientation of the α-Al matrix is represented in the unit triangle. 

The white and black particles were distributed in the α-Al matrix (Figure 2a), and they correspond to the Mg_2_Si phase with dark contrast [17] and the β-AlFeSi (τ_6_-Al_9_Fe_2_Si_2_) phase with bright contrast [18] formed in the α-Al matrix [12]. The orientation map indicates that the α-Al matrix exhibits relatively equiaxed grains with a mean size ranging from approximately 5 to 20 μm (Figure 2b). 

**Figure 3:**
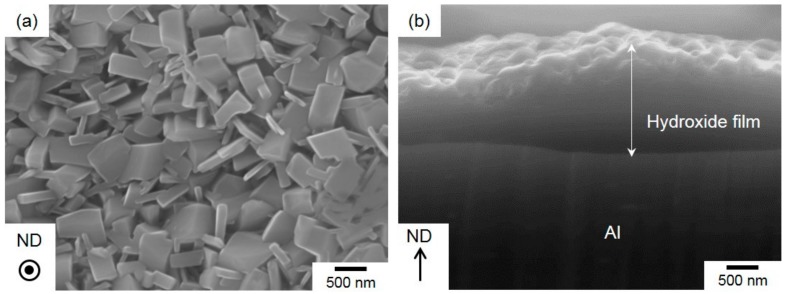
High-magnification SEIs showing the (**a**) top view and (**b**) cross-sectional view of the hydroxide films on the steam-coated Al-alloy sheets.

Figure 3 presents the top and cross-sectional views of the hydroxide film in the steam-coated specimen. 

The film consisted of numerous polyhedral particles approximately 300 nm in size (Figure 3a), which corresponds well to previous reports on the surface morphologies of hydroxide films [19,20,21]. Cross-sectional observation revealed that the thickness of hydroxide film was approximately 2 μm, noting that the top surface shows an irregular shape and bottom had a flat interface on the Al-alloy substrate (Figure 3b).

**Figure 4:**
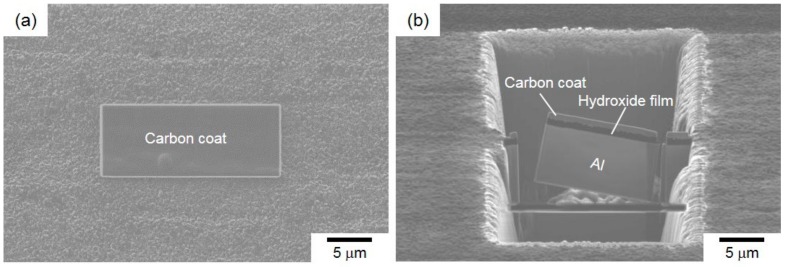
Preparation process of site-specific TEM samples of Al-alloy sheet (containing a hydroxide film) using a focused ion beam (FIB) pickup technique: (**a**) a deposited carbon layer on the surface of hydroxide film on the steam-coated Al-alloy sheet; (**b**) a cross-section sample milled out by FIB.

Figure 4 presents the preparation process of the TEM sample using a FIB pickup technique for observation of the hydroxide film. The sample surface was ion-milled by using FIB operated at an accelerating voltage of 30 kV and various probe current values ranging from 50 to 5000 pA. A deposited protection layer (carbon layer) was used to avoid Ga ion damage to the surface of hydroxide film (Figure 4a).

**Figure 5:**
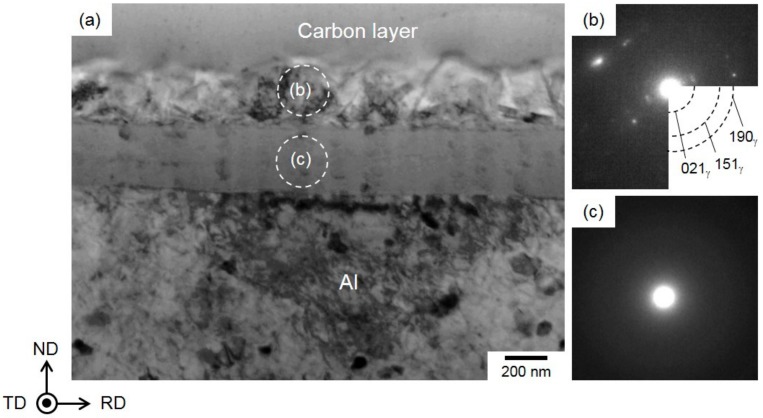
(**a**) TEM bright-field image and (**b,c**) selected-area electron diffraction (SAED) patterns for (**b**) the top γ-AlO(OH) layer and (**c**) the bottom continuous amorphous layer of hydroxide film that was formed on the steam-coated Al-alloy sheet.

A thin cross-section sample with the thickness of less than 100 nm (containing with hydroxide film) was milled out from Al-alloy sheet by FIB (Figure 4b). This sample was picked up using a glass probe and then put on the Cu-grid mesh coated with an organic film. The obtained TEM bright-field images and selected-area electron diffraction (SAED) patterns of local hydroxide films are presented in Figure 5. 

**Figure 6:**
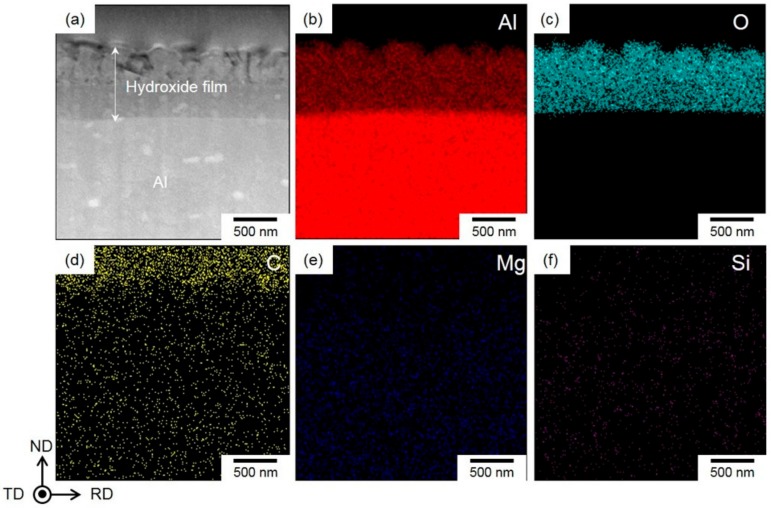
(**a**) Scanning TEM (STEM)–high-angle annular dark field (HAADF) image showing the hydroxide film formed on the steam-coated Al-alloy sheet and the corresponding (**b**) Al; (**c**) O; (**d**) C; (**e**) Mg and (**f**) Si element maps by energy dispersive X-ray spectroscopy (EDS) analysis.

The hydroxide film shows a dual-layer structure with the two layers having almost the same thickness (Figure 5a). The γ-AlO(OH) phase in the upper layer was determined by the SAED pattern (Figure 5b). The captured diffraction spots obviously indicate a polycrystalline γ-AlO(OH) phase composed of a number of fine γ crystals with a mean size below 300 nm (Figure 5a). Note that no clear diffraction spots were detected in the SAED pattern of the lower layer within the hydroxide film (Figure 5c), indicating that this layer adjacent to the Al-alloy substrate was amorphous. The EDS composition analyses revealed that the Al and O elements were enriched inside the hydroxide film (Figure 6a–c), and there was no clear difference in their contents between the upper and lower layers. Hence, Mg- and Si-alloy elements were not enriched in the hydroxide film, indicating that the composition of the second phases (Mg_2_Si phase and β-AlFeSi phase) did not obviously affect the initial formation of hydroxide film.

These microstructural characteristics indicated that the hydroxide film formed had a dual-layered structure, composed of an outer γ-AlO(OH)-phase layer on the surface and a continuous inner amorphous layer on the Al substrate.

### 3.2. In Situ Observation of Crack Initiation and Propagation of Hydroxide Film 

**Figure 7:**
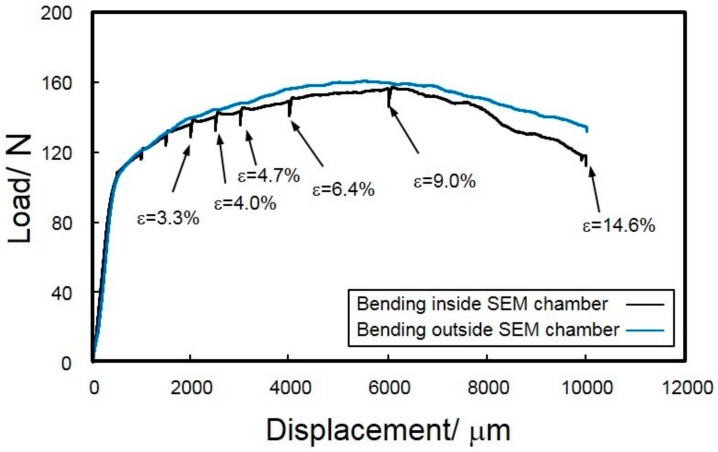
Load-displacement curves of the steam-coated Al-alloy sheets obtained from 4-points bending tests.

Figure 7 presents two representative load-displacement curves of steam-coated specimens in the bending tests.

**Figure 8:**
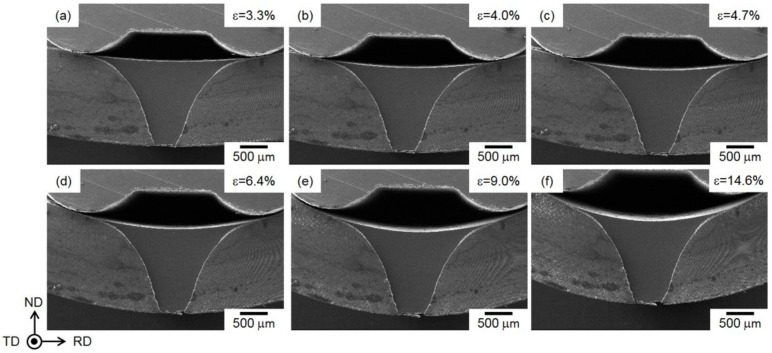
SEIs showing the bent steam-coated specimens, in which the applied strain was measured to be approximately (**a**) 3.3%, (**b**) 4.0%, (**c**) 4.7%, (**d**) 6.4%, (**e**) 9.0%, and (**f**) 14.6%.

Stress drops are often observed in the flow curve of bending tests conducted in the SEM chamber, when the bending must be interrupted to allow minute SEM observations of microstructure. These stress drops correspond to the stress relaxations in the specimen during the interrupting bending test for in situ SEM observations. Nevertheless, the flow curves exhibit a yielding and subsequent strain hardening behavior and agree well with the curve from uninterrupted bending tests conducted outside the SEM chamber. Low-magnification in situ SEIs obtained during the bending test are displayed in Figure 8. The specimen was uniformly deformed with an increasing punch stroke (displacement). For the specimens deformed at different displacements, the radius (*R*) of curvature was experimentally measured using the in situ observed SEIs (Figure 8). Note that the observed V-shaped area on the cross section of the specimen corresponds to the observed ion-polished surface by the argon-ion beam cross-section polisher (CP).

The measured *R* values can be used to calculate the macroscopic strains (*ε*) of the specimen following Equation (1) (as schematically illustrated in Figure 1(d). The calculated *ε* ranged from 3.3% to 14.6% for different displacements, as labeled on the flow curve in Figure 7.

**Figure 9:**
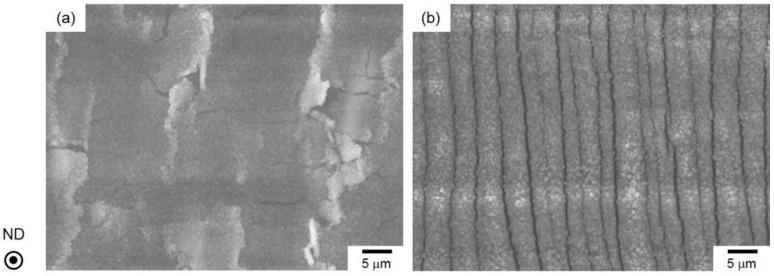
Top-view SEIs showing the fracture morphology in the (**a**) compressively and (**b**) tensile-strained surfaces of hydroxide films on the steam-coated 6061 alloy sheet with the higher applied strain of 14.6%.

Figure 9 displays representative SEIs of the hydroxide film after the specimen was deformed at a large displacement of 10 mm. 

The applied strain was calculated as 14.6%. The fracture morphology depends on the strain state of the surface. Local delamination occurs for the hydroxide film on the compressively strained surface (Figure 9a), while parallel cracks with a spacing of approximately 2.5 μm were found on the tensile-strained surface (Figure 9b). These results correspond well to the previous study about fracture morphologies of hydroxide film on the Al-alloy sheet [12]. It is noteworthy that no peeled parts of a lower amorphous layer on the Al substrate were observed on either surface, suggesting the hydroxide film on the Al-alloy substrate has a higher cohesive strength. 

**Figure 10:**
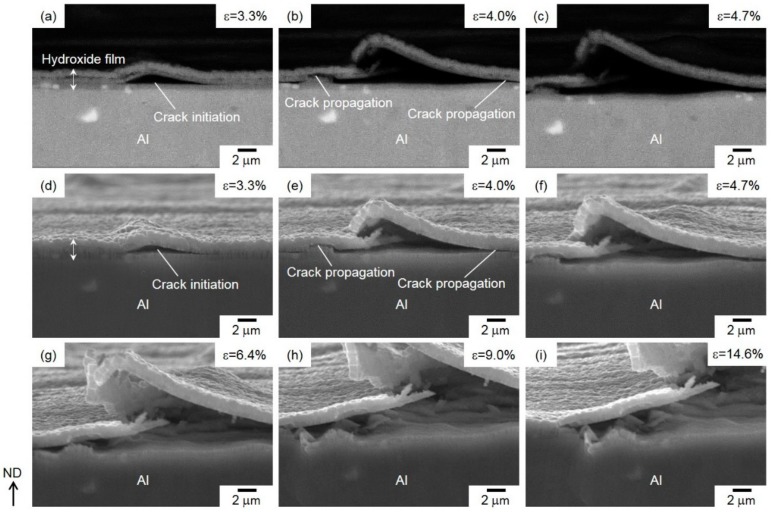
(**a–c**) High-magnification backscattered images (BEIs) and (**d–i**) SEIs showing cracks initiated and propagated on the compressively strained surface of hydroxide films formed on Al-alloy sheet, under a growing macrostrain of approximately (**a,d**) 3.3%, (**b,e**) 4.0%, (**c, f**) 4.7%, (**g**) 6.4%, (**h**) 9.0%, and (**i**) 14.6%.

Figure 10 presents high-magnification, in situ observed backscattered images (BEIs) and SEIs, in order to show the cracking process of the hydroxide film on the compressively strained surface of the bent specimen.

A selected area of the deformed specimen was captured using both BEI (Figure 10a–c) and SEI (Figure 10d–i) modes. These images show that a lateral crack was initiated inside the hydroxide film when the specimen was under a macroscopic strain of *ε* = 3.3% (Figure 10a,d). The BEI (Figure 10a) clearly demonstrates that the crack was initiated within the lower amorphous layer. This lateral crack propagated not only inside the amorphous layer but also along the interface between the lower amorphous layer and the upper γ-AlO(OH) layers (Figure 10b,e). In addition, note that the cracking process of hydroxide film was not affected by the second β-AlFeSi phase with bright contrast. The propagated lateral crack appeared to distort the hydroxide film, resulting in the fracture of the upper γ-AlO(OH) layer (Figure 10c,f) by applying a higher strain of *ε* = 4.7%. The fracture process proceeded under further strain, leading to the delamination of the upper γ-AlO(OH) phase and top portions of the amorphous layer (Figure 10g–i). This result corresponds to the delamination of the hydroxide film that occurred locally along the surface normal (Figure 9a). For in situ SEM observation, on the other hand, it demonstrated that the lower amorphous layer did not peel off from the Al-alloy substrate even under a high applied strain of 14.6% (Figure 10i).

**Figure 11:**
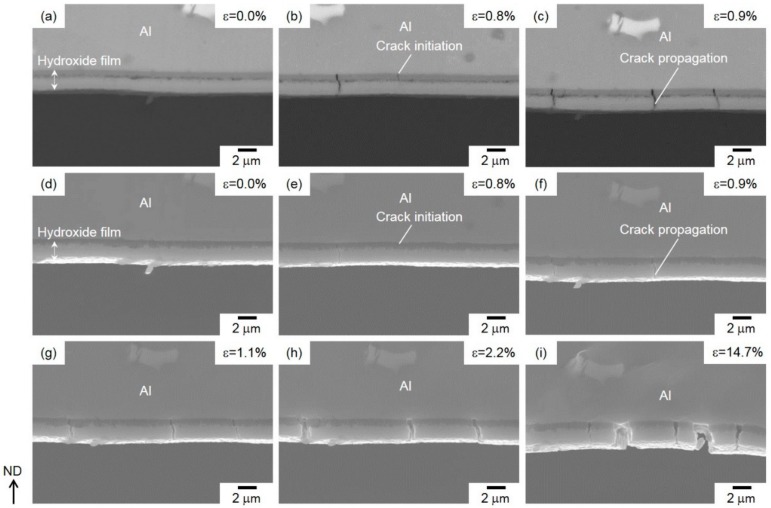
(**a–c)** High-magnification BEIs and (**d–i**) SEIs showing cracks initiated and propagated in the tensile-strained surface of hydroxide films on the Al-alloy sheet with increasing applied macrostrains of approximately (**a,d**) 0.0%, (**b,e**) 0.8%, (**c, f**) 0.9%, (**g**) 1.1%, (**h**) 2.2%, and (**i**) 14.7%.

Figure 11 displays the high-magnification in situ observed BEIs (Figure 11a–c) and SEIs (Figure 11d–i) in the hydroxide film on the tensile-strained surface of the bent specimen. 

No cracks were found inside the hydroxide film before the bending test (Figure 11a,d), while at a relatively low *ε* = 0.8%, a couple of vertical cracks were initiated in the lower amorphous layer (Figure 11b,e). The initiated vertical cracks penetrate the upper γ-AlO(OH)-phase layer along the surface normal (Figure 11c,f). Under further tensile loading, different vertical cracks were initiated and passed through both the upper and lower layers (Figure 11g). The opening of the propagated cracks was clearly observed by further straining (Figure 11h,i), resulting in a constant spacing of the cracks observed along the surface normal (Figure 9b). These vertical cracks were arrested around the Al-alloy substrate, where removal of a lower amorphous layer on the Al substrate was not found, even on the highly strained surface (Figure 11i), implying a high cohesive strength of the lower amorphous layer on the Al-alloy substrate. 

## 4. Discussion

In the present study, we observed the bending-induced fracture process of hydroxide film in steam-coated Al-alloy sheets at the elevated temperature of 220 °C. With a dual-layered structure, the film comprised an amorphous layer on inside and an γ-AlO(OH)-phase layer on the outside (Figure 5). Bending tests revealed that the fracture morphology of the hydroxide film depends on the applied strain state (Figure 9). In situ SEM observation (Figure 10 and Figure 11) demonstrated that the cracks are preferentially initiated inside the lower amorphous layer during sample bending. A number of vertical cracks occurred on the hydroxide film on the tensile-strained surface, but the hydroxide film was not removed from the Al sheet (Figure 11). On the compressively strained surface, lateral cracks propagate into the lower amorphous layer or along its interface with the upper γ-AlO(OH) layer (Figure 10). The propagation of lateral cracks could be responsible for the local delamination observed on these film surfaces (Figure 9a). The preferential cracking might be due to the peculiar dual-layered structure of the hydroxide film. In compressive deformation (Figure 10), concentrated stress/strain might be localized near the interface between the amorphous layer and Al-alloy substrate, resulting in crack initiation in the amorphous layer.

**Figure 12:**
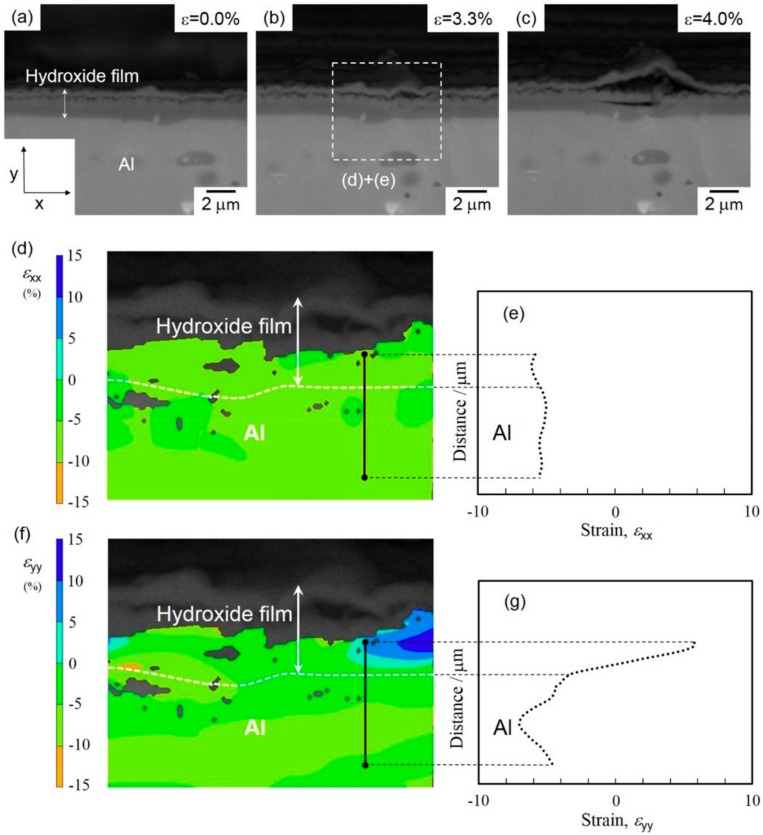
(**a–c**) High-magnification BEIs showing cracks initiated and propagated in the compressively strained surface of hydroxide films on the Al-alloy sheet under an increasing compressive strain. The applied macrostrain was calculated to be approximately (**a**) 0.0%, (**b**) 3.3%, and (**c**) 4.0%. The corresponding (**d,f**) strain distribution maps for the selected area in (**b**) and (**e,g**) strain-distribution profiles for the selected line containing the initiated cracks in hydroxide film of (**d,e**) *ε*_xx_ and (**f,g**) *ε*_yy_ (obtained by digital image correlation (DIC) analysis) along the x and y directions.

To confirm the speculated mechanism, the strain distribution around the hydroxide film was quantified by DIC analysis of the in situ observed SEM images showing the fracture process (as displayed in Figure 10). The results from the DIC analysis are summarized in Figure 12. 

No cracks were observed inside the hydroxide film before the bending test (Figure 12a). At a low strain of 3.3%, one crack appeared in the outer γ-AlO(OH) layer, but there was none in the lower amorphous layer (Figure 12b). Further straining (*ε* = 4.0%) led to crack initiation in the amorphous layer and the associated delamination of the γ-AlO(OH) layer (Figure 12c). In order to investigate the effect of concentrated strain on crack initiation, the strain distribution around the hydroxide film deformed at *ε* = 3.3% was quantified by DIC analyses. The visualized strain maps of *ε*_xx_ and *ε*_yy_ and the corresponding strain profiles are presented in Figure 12d–g, with the horizontal and vertical directions defined as x and y directions, respectively. In the distribution map of the *ε*_xx_ component (Figure 12d), negative values from −5% to −10% were found in all analyzed areas in the amorphous layer (Figure 12b). The strain profile in Figure 12e along a line across the amorphous layer (as indicated in Figure 12d) shows an almost constant strain of approximately −6%. These results obviously indicate a uniform compressive strain (along the x direction) applied by bending deformation. In contrast, the distribution of the *ε*_yy_ component in the hydroxide film was inhomogeneous: negative values of *ε*_yy_ (compressive strain) were found in the Al-alloy substrate, whereas positive values (tensile strain) were detected inside the amorphous layer (Figure 12f). In other words, the sign of *ε*_yy_ changes near the interface between Al-alloy substrate and the amorphous layer, as presented by the line profile of *ε*_yy_ strain in Figure 12g. Importantly, it was found that the compressive strain (along the surface normal) was applied in the Al-alloy substrate, whereas the tensile strain (along the surface normal) was localized at the neighbor amorphous layer on the compressively strained surface of specimens deformed by bending. The site of localized tensile strain corresponds well to that of crack initiation (Figure 12c). Thus, the variation of strain state around the amorphous layer could facilitate the opening of cracks near the amorphous layer/Al-alloy interface. Since the Al substrate is more ductile than the adjacent amorphous layer, cracks are preferentially initiated inside the latter. This varied strain state around the hydroxide film might be responsible for a difference in elastic deformation between the amorphous phase and α-Al (fcc) matrix. Overall, the present strain-distribution analysis demonstrates that the concentrated tensile strain along the surface normal (positive *ε*_yy_) at some parts of the amorphous layer contributed to crack initiation in this layer (as presented in Figure 10). 

It should be noted that no crack was found at the interface of the amorphous layer with the Al-alloy substrate. As a result, the anticorrosion hydroxide film was not removed from the Al sheet, even after strong deformation by bending. Such good adhesion of the amorphous layer could prevent delamination of the hydroxide film, and the resulting continuous amorphous layer could maintain the corrosion resistance. Therefore, the superior corrosion resistance of the steam-coated Al-alloy parts is probably only affected slightly by the plastic-forming processes. However, the detailed mechanism responsible for the high adhesive strength of the Al-containing amorphous layer with the α-Al matrix is still unclear, although the interfacial properties could be an important factor. Atomic-level characterizations of this interface will be required to further reveal the adhesion mechanism.

## 5. Summary

Using in situ SEM observations, we investigated the crack initiation and propagation inside the hydroxide film with a dual-layered structure (an amorphous layer on the inside and an polycrystalline γ-AlO(OH) layer on the outside) formed on Al alloy by steam coating during specimen bending in the SEM chamber. The key results are summarized as follows:

On the compressively strained surface, lateral cracks were preferentially initiated inside the lower amorphous layer, owing to the concentration of tensile strain along the surface normal in some parts of that layer. The lateral cracks propagated either inside the lower amorphous layer or into the interface between the two layers, leading to local delamination of the hydroxide film on the Al-alloy sheet.

On the tensile-strained surface, vertical cracks are preferentially initiated inside the inner layer and penetrate the outer layer. These cracks are opened under further strain, but removal of the hydroxide film did not occur. 

No cracks were found at the interface between the amorphous layer and the Al-alloy substrate, and as a result the anticorrosion hydroxide film was not removed from the Al sheet, even during strong bending. The high adhesive strength could prevent reduction in the corrosion resistance when the steam-coated Al-alloy parts undergo plastic-forming processes.
